# Draft Genome Sequences of Two *Paenibacillus* Isolates from Goose and Chicken Gastrointestinal Material

**DOI:** 10.1128/MRA.00013-20

**Published:** 2020-03-05

**Authors:** Jitendra Keshri, Rocio Ramirez, Molly K. Svendsen, Haley R. Keillor, Madeline L. Moss, Haley J. Jordan, Kristina M. Smith, Patrick N. Ball, Bruce S. Seal, Mark E. Berrang, Brian B. Oakley

**Affiliations:** a College of Veterinary Medicine, Western University of Health Sciences, Pomona, California, USA; b Graduate College of Biomedical Sciences, Western University of Health Sciences, Pomona, California, USA; c Biology Program, Oregon State University Cascades, Bend, Oregon, USA; d USDA—Agricultural Research Service, U.S. National Poultry Research Center, Athens, Georgia, USA; Loyola University Chicago

## Abstract

Here, we present the draft genome sequences of two *Paenibacillus* strains, An7 and USDA918EY, isolated from goose feces (Bend, OR, USA) and chicken ceca (Pomona, CA, USA), respectively. These data may assist with analyses of microorganisms associated with free-ranging and commercial avian species.

## ANNOUNCEMENT

The genus *Paenibacillus* was first proposed in 1993 for the monophyletic lineage of endospore-forming bacteria previously assigned to the genus *Bacillus* (1–3). A total of 242 species belonging to the genus *Paenibacillus* have been reported as of 2019 (http://www.bacterio.net/paenibacillus.html). We isolated a species belonging to the genus *Paenibacillus*, designated *Paenibacillus* sp. strain An7, from the feces of resident Canada goose (Branta canadensis) populations in Bend, Oregon, utilizing brucella agar with blood and vitamin K-hemin (BD) under anaerobic conditions (Oxoid, AnaeroGen). Another isolate of this genus was recovered from the ceca of a broiler chicken (Gallus domesticus) in Pomona, California, by using an aerobic environment on tryptose sulfite cycloserine agar and was designated strain USDA918EY. DNA was extracted from overnight cultures with the QuickExtract bacterial DNA extraction kit (An7) or the Mo Bio UltraClean microbial DNA isolation kit (USDA918EY). The genomes of both strains were sequenced using the Illumina NovaSeq platform through the Institute for Integrative Genome Biology (IIGB) Genomics Core at the University of California, Riverside.

Libraries with a fragment length of 150 bp were constructed using the PlexWell 96 library preparation kit for Illumina sequencing platforms (seqWell, Inc., Beverly, MA, USA), and totals of 1,678 and 10,113 Mb of paired-end (150-bp) reads were generated for strains An7 and USDA918EY, respectively. Quality filtering of the reads was performed using Trimmomatic v0.39 (4). High-quality reads were used for *de novo* genome assembly with SPAdes v3.12.0, using the careful assembly method (5). Scaffolds were filtered for a minimum 200-bp read length and 30× coverage. The quality of the subsequent assemblies was assessed using QUAST (6). Genome completeness was found to be 93% and 99.8% for strains An7 and USDA918EY, respectively, using the CheckM lineage workflow (7). A search for similar genomes using the Mash/Minhash algorithm (8) showed the nearest genomes to strains An7 and USDA918EY to be *Paenibacillus* sp. strain TCA20 (accession no. BBIW00000000; Mash distance, 24%) and *Paenibacillus* sp. strain P1XP2 (accession no. JRNV00000000; Mash distance, 11%), respectively. The species identification tool SpecI (9), based on 40 universal single-copy marker genes, confirmed that neither genome could be assigned to any species cluster. The assembled genomes were annotated using the IMG/MER pipeline (10), and the average coverages were calculated using the BBMap tool. The assembly and annotation metrics are presented in Table 1.

**TABLE 1:** Assembly and annotation metrics

Statistic	Data for:	
	An7	USDA918EY
No. of quality-filtered paired-end reads	1,825,939	11,546,162
No. of scaffolds	131	93
*N*_50_ (bp)	179,529	371,097
Largest scaffold length (bp)	659,161	944,192
Assembly length (bp)	4,558,425	6,732,310
Avg coverage (×)	93	442
G+C content (%)	41.48	51.98
No. of rRNA genes	10	10
No. of tRNA genes	47	49
No. of functional protein coding genes	3,269	4,736
No. of hypothetical protein coding genes	1,092	1,520
No. of genes associated with KEGG pathways	1,132	1,500
No. of genes associated with KEGG orthology	2,350	2,732
GenBank accession no.	WTWX00000000	WTWY00000000
SRA accession no.	SRR10742426	SRR10742606

Based on the compare genome analysis function in IMG, which uses pairwise alignment, the pairwise maximum average nucleotide identity (ANI) of strain An7 with known genomes of *Paenibacillus* is 74.03% with *Paenibacillus* sp. TCA20 (accession no. BBIW00000000) (aligned fraction, 54%). The maximum ANI of USDA918EY with known genomes of *Paenibacillus* is 87.6% with *Paenibacillus* sp. P1XP2 (accession no. JRNV00000000) (aligned fraction, 53%). The ANI between the two isolates is only 69.5%, suggesting that strains An7 and USDA918EY are distantly related to each other. Further comparative analyses are now possible and will provide insights into the functionality of these strains.

### Data availability.

The draft genome assemblies and raw sequences of these strains have been deposited in DDBJ/ENA/GenBank under the accession numbers listed in Table 1.
